# Food Insecurity among Older Aboriginal and Torres Strait Islanders

**DOI:** 10.3390/ijerph15081766

**Published:** 2018-08-17

**Authors:** Jeromey B. Temple, Joanna Russell

**Affiliations:** 1 Demography and Ageing Unit, Melbourne School of Population and Global Health, University of Melbourne, Melbourne 3010, Australia; 2 School of Health and Society, University of Wollongong, Wollongong 2522, Australia; jrussell@uow.edu.au

**Keywords:** food insecurity, food security, Indigenous population, ageing, Indigenous

## Abstract

It is well established that Indigenous populations are at a heightened risk of food insecurity. Yet, although populations (both Indigenous and non-Indigenous) are ageing, little is understood about the levels of food insecurity experienced by older Indigenous peoples. Using Australian data, this study examined the prevalence and correlates of food insecurity among older Aboriginal and Torres Strait Islanders. Using nationally representative data, we employed ordinal logistic regression models to investigate the association between socio-demographic characteristics and food insecurity. We found that 21% of the older Aboriginal and Torres Strait Islander population were food insecure, with 40% of this group exposed to food insecurity with food depletion and inadequate intake. This places this population at a 5 to 7-fold risk of experiencing food insecurity relative to their older non-Indigenous peers. Measures of geography, language and low socio-economic status were highly associated with exposure to food insecurity. Addressing food insecurity offers one pathway to reduce the disparity in health outcomes between Aboriginal and Torres Strait Islanders and non-Indigenous Australians. Policies that consider both remote and non-remote Australia, as well as those that involve Aboriginal people in their design and implementation are needed to reduce food insecurity.

## 1. Introduction

Like many Indigenous populations, the Aboriginal and Torres Strait Islander population in Australia has considerably poorer health outcomes when compared to their non-Indigenous peers, experiencing a range of health conditions at an earlier age of onset and considerably lower life expectancy [1,2,3,4]. The health issues facing many older people is an issue of increasing importance given persisting inequalities in socio-economic outcomes, as well as the ageing of this population [5]. In the 10 years to 2026, the population of Aboriginal and Torres Strait Islanders aged 45 and over is projected to increase by 35%, accounting for 22% of this population [6]. 

Importantly, many of the health conditions prevalent among older Aboriginal and Torres Strait Islanders are preventable, notwithstanding the levels of socio-economic disadvantage faced by this population. The Australian Institute of Health and Welfare (2016) estimates that the burden of disease in the Aboriginal population is 2.3 times of that experienced by non-Indigenous Australians [2]. Through reducing exposure to modifiable risk factors, such as tobacco and alcohol use and poor dietary behaviors, they estimate that about half of the difference in disease burden between Aboriginal and non-Indigenous Australians could be removed. In total, poor dietary behaviors were estimated to account for 10% of the total burden of disease faced by Aboriginal and Torres Strait Islanders.

An important proximate determinant and risk factor of poor nutrition outcomes is food insecurity. Food insecurity refers to the “limited or uncertain availability of nutritionally adequate and safe foods or limited or uncertain ability to acquire acceptable foods in socially acceptable ways” [7]. A growing number of studies have shown that Indigenous populations around the world are at a heightened risk of food insecurity [8,9,10,11,12]. For example, Canadian research has shown that Aboriginal people living on reservations were at 2.6 times the risk of being food insecure when compared to their non-Indigenous peers [8]. Research from the USA shows that levels of food insecurity experienced by American Indians are double that reported by non-Indigenous people [9]. Within the American population, food insecurity rates among the Navajo Nation were the highest reported within the USA to date, with just under 80% of this population experiencing some form of food insecurity [10]. Evidence from Australia shows that about 22% of Aboriginal and Torres Strait Islanders were exposed to food insecurity compared with 4% of non-Indigenous Australians [13]. 

Despite evidence of heightened risk of food insecurity within Indigenous populations, little is known about the prevalence and correlates of food insecurity in the older Aboriginal and Torres Strait Islander population. However, studies have examined food insecurity among the older non-Indigenous population, concluding the strength of socio-economic factors in explaining food insecurity in later life [14,15,16]. The aim of this study is to address this research gap through an examination of food insecurity among older Aboriginal and Torres Strait Islanders. Firstly, we assess the prevalence of food insecurity in Aboriginal and Torres Strait Islander older adults and secondly, we examine the risk factors for food insecurity in this population. We conclude with a discussion of the implications of our findings. 

## 2. Materials and Methods 

### 2.1. Survey Data

Data for this study are from the 2012–2013 Australian Aboriginal and Torres Strait Islander Nutrition and Physical Activity Survey (NATSINPAS) conducted by the Australian Bureau of Statistics (ABS) from August 2012 to July 2013 [17]. The survey is based on a sample of 2900 private dwellings across Australia, with a response rate of 79% (3661 households were approached). Within each dwelling, one adult (aged 18 and over) and one child (where applicable) were randomly selected for interview. The survey excluded those living in non-private dwellings such as hospitals, nursing homes and hotels. The survey also excluded all non-Indigenous persons including non-Indigenous born Australians, non-Australian diplomats, and overseas visitors. 

An important advantage of the NATSINPAS is the geographic coverage of the survey, including remote and non-remote areas as well as discrete Aboriginal and Torres Strait Islander communities. Remote areas include very remote Australia and remote Australia under the Australian Statistical Geography Standard [18]. Non-remote areas include those residing in major cities, inner or outer regional Australia. The final sample included data on 1792 individuals living in non-remote areas and 2317 individuals in remote areas in Australia. ABS interviewers conducted face to face interviews, collecting information on health, nutrition and characteristics of the household and dwelling. 

NATSINPAS was part of the larger Australian Aboriginal and Torres Strait Islander Health Survey (AATSIHS). The sampling design of NATSINPAS sought to provide reliable estimates at the national level, as well as for remote and non-remote Australia. The in-scope population was divided into two broad populations. The first group included those living in discrete Aboriginal and Torres Strait Islander communities in remote Australia, defined as the ‘community frame’. The second group included the remainder of the in-scope population, referred to as the ‘non-community frame’. The Indigenous Community Frame (ICF) was used to target respondents for the community frame, and was built from a combination of Census data as well as data from the Discrete Indigenous Communities Database. The non-community frame was formed using Census data at a low geographic level (SA1). For some States and Territories, the non-community sample was disaggregated into non-remote and remote non-community areas. Survey weights at the person level were calculated by the ABS to calibrate the probability of selection into the survey relative to estimated resident population counts (less those in non-private dwellings).

Data from NATSINPAS were collected under the Census and Statistics Act 1905. As NATSINPAS included a biomedical component, ethics approvals were sought at both the national level (by the Australian Government Department of Health and Ageing) and at the state and Territory level (by a range of medical and Aboriginal ethics committees). Data for this study were made available by the ABS through the Universities of Australia agreement. NATSINPAS survey data are available to registered users of ABS microdata. 

### 2.2. Measurement

In this study, we consider the prevalence and risk factors for food insecurity among older Aboriginal and Torres Strait Islanders aged 45 years and over (*n* = 1062). Defining the sample aged 45 years and over as ‘older’ is common in studies of ageing among Aboriginal and Torres Strait Islander people for several reasons [4,19,20]. Firstly, there is a considerable gap in life expectancy of about one decade between Aboriginal and non-Indigenous Australians, reducing the proportion of the population living into advanced old age [3,21]. Second, many conditions and comorbidities as well as frailties commonly associated with aging are early onset in this population [2,3,22]. Third, in recognition of the above two points, government programs such as those governing access to specific aged care services are available to Aboriginal and Torres Strait Islanders from earlier ages when compared to non-Indigenous Australians.

The measurement of food insecurity in NATSINPAS consists of two questions. The household spokesperson (adult aged 18 or over) was asked, “In the last 12 months was there any time when you (or members of this household) ran out of food and couldn’t afford to buy more?” A follow up question was asked, “When this happened, did you (or members of this household) go without food?” Following other Australian studies, we use these two questions to create a variable which views food insecurity on a continuum [23]. Those answering ‘no’ to the first question are coded as ‘food secure’. Those who answer ‘yes’ to the first question, but ‘no’ to the second are coded as ‘food insecure—food depletion’. That is, they ran out of money for food, but did not go without food. The final group, ‘food insecure—food depletion and inadequate intake’ are those who ran out of money for food and went without food consequently. Although there are a number of limitations to this measure (as discussed in Section 4.4), this is the most detailed measure of financially attributable food insecurity that is collected by the ABS on an irregular basis. 

### 2.3. Statistical Model

To model the association between socio-economic characteristics and food insecurity, we utilized Stata 14.0. As the dependent variable is ordinal, standard logistic regression is inappropriate. We utilized ordered logistic regression fitted by maximum likelihood [24]. Initial variable selection was informed by the growing literature on food insecurity among older Australians [14,15,16]. Variables entered the regression model and improvement to model fit assessed using the Bayesian Information Criteria following Raftery’s (1995) procedure [25]. With the full model specified, we check the conditioning of the matrix of independent variables to investigate any collinearity influence [26]. The condition numbers were very small providing support for the model specification. An important assumption underlying ordinal logistic regression is the parallel lines or proportional odds assumption. This assumption states that the coefficients of variables associated with the probability of food security versus food insecurity with depletion and food security versus depletion and inadequate intake are constant. We follow Brant’s (1990) procedure and the non-significant test result provides strong support for modeling the severity of food insecurity within an ordinal framework [27]. 

#### Independent Variables

Informed by the literature on food insecurity in Australia and using model selection techniques outlined above, variations in food insecurity were identified by a range of socio-economic characteristics. The final variables included in the model were:

- Age: Measured in aggregate categories (45–54, 55–64, 65–74, 75+)
- Marital Status: Married or not married. Unfortunately, more detailed items such as widowed, never married or separated were unavailable on the data set.
- Gender: Male or Female.
- Household Size: Categorized as 1, 2, 3 or 4, 5 or more.
- Household Composition: Whether the household includes Aboriginal and Torres Strait Islander members only, or with non-Indigenous members also present.
- Indigenous Language Speaking: Whether the persons speaks an Australian Indigenous language.
- Remoteness: Whether the household resides in remote or non-remote parts of Australia, as defined by the Australian Statistical Geography Standard (ASGS).
- Household Income: The measure of household income provided by the ABS was equivalized household income (adjusted or equivalized using an equivalence scale) and collapsed into deciles. This adjusted form of household income allows for welfare and financial wellbeing comparisons between households of different sizes and compositions.
- Self-Reported Health: A dichotomous variable indicating whether the person self-reported their health as being excellent or good versus fair or poor.

## 3. Results

### 3.1. Prevalence of Food Insecurity

Table 1 displays the weighted estimates of the severity of food insecurity among Aboriginal and Torres Strait Islanders aged 45 and over. In 2012–2013, approximately 21% of this population reported being food insecure. Of this group of food insecure persons, about 41% reported both food depletion and inadequate intake, that is, they ran out of money and went without food consequently. This group accounted for about 8% of the Aboriginal and Torres Strait Islander population aged 45 years and over. 

There is considerable socio-economic and demographic variation in exposure to food insecurity among older people (Table 1). Important to this analysis of food insecurity among older Aboriginal and Torres Strait Islanders is Indigenous language speaking, living in a remote area, and Indigenous household composition. Interestingly, we observe differences in exposure to food insecurity by discrete categories of English language speaking and geography. In this sample, almost all respondents in non-remote areas speak English in the household. Approximately 19% of this group were food insecure, as were those who spoke English in remote areas of Australia. However, for those who speak Indigenous languages residing in remote areas, the prevalence of food insecurity was almost double (about 37%). Indeed, 12% of Indigenous language speakers in remote areas were severely food insecure (or one third of all food insecure people).

Similarly, strong differences in food insecurity by Aboriginal household composition are observed. Those living in a household with both Aboriginal and non-Indigenous members have a food insecurity prevalence of about 7%, compared with 28% of those in Aboriginal only households. Overall, we identify several population sub-groups with a food insecurity prevalence rate of around 30%: Indigenous speakers in remote communities (37%), Aboriginal and Torres Strait Islander only households (28%), smokers (31%), persons in households with a large number of occupants (32%), non-married females (35%), and income earners in the lowest centile (31%).

Although these descriptive results indicate significant differences in exposure to food insecurity by socio-economic characteristics, it is important to control for confounding effects to measure the association between each covariate and the probability of food insecurity. 

### 3.2. Regression Results

With a range of demographic and economic controls, English speaking and remoteness remain strong predictors of food insecurity (Table 2). Those speaking Indigenous languages were about 57% more likely to experience food insecurity compared with those in non-remote areas (OR 1.57 95% CI: 1.01–2.44). Again, there was no difference in food insecurity between English speakers in remote and non-remote areas (OR = 0.99 *p* < 0.10). In a similarly sized effect, households with both Aboriginal and non-Indigenous residents were about 60% less likely to experience food insecurity compared with Aboriginal only households, even with controls for geography (OR 0.4 95% CI: 0.22, 0.69).

Non-smokers remained 39% less likely to experience food insecurity (OR 0.61 95% CI: 0.44, 0.84) and persons in large households were at a high risk, particularly those in household with 5 or more occupants (OR 2.64 95% CI: 1.28, 5.46). Those in the top 20% of the income distribution were about 88% less likely (OR 0.12 95% CI: 0.02, 0.95) to suffer food insecurity and those in the 60–80 percentile were about 76% less likely (OR 0.24 95% CI: 0.08, 0.71) when compared to lowest income earners. 

Gender and social marital status also remains significant in the regression models. Compared to unmarried females, married females were about 48% less likely to experience food insecurity (OR 0.52 95% CI: 0.30, 0.89). Married males were 38% less likely to experience food insecurity relative to non-married females, but the estimate is only significant at the 90% level. 

## 4. Discussion

Although several Australian studies have examined food insecurity in the older population, little is known about the prevalence and risk factors of food insecurity among older Aboriginal and Torres Strait Islanders specifically. In this paper, we have used nationally representative survey data to measure the prevalence and correlates of food insecurity among Aboriginal and Torres Strait Islanders aged 45 and over.

### 4.1. High Prevalence of Food Insecurity among Older Aboriginal and Torres Strait Islanders

Firstly, we find the prevalence of food insecurity among older Aboriginal and Torres Strait Islanders is high with about 21% reporting exposure, and 41% of this group reporting food depletion and inadequate intake. These estimates are in line with published results from the ABS showing that about 22% of Aboriginal and Torres Strait Islanders experience food insecurity compared to 4% of non-Indigenous Australians (ABS, 2015). The prevalence of food insecurity among older non-Indigenous Australians using a similar measure has previously been reported at 3% [14,28]. That is, older Aboriginal and Torres Strait Islanders are at about a 5–7 fold risk of food insecurity relative to their non-Indigenous peers.

This finding is important for several reasons. There is now a significant body of evidence on the implications of food insecurity for individual health and wellbeing. International studies show exposure to food insecurity is associated with symptoms of depression and anxiety, multimorbidity, lower levels of self-reported health status, lower nutrient diets, a greater likelihood of reporting social isolation, long standing health problems and activity limitations, and a greater likelihood of reporting heart disease, diabetes, and high blood pressure [29,30,31,32,33,34,35,36,37]. Among non-Indigenous Australians, food insecurity has also been shown to be associated with self-reported depression, reduced quality of life and poor diet quality [14,23,38]. More broadly, food insecurity may be related to decreased productivity and social interaction and contribute to increased economic inequality [39].

The high prevalence of food insecurity among older Aboriginal and Torres Strait Islanders is also important in the context of the gap in health outcomes that persists between Aboriginal and non-Indigenous Australians. ‘Closing the Gap’ is an Australian Government strategy whereby a key goal is to improve the health outcomes of Aboriginal and Torres Strait Islander populations. Specifically, the strategy seeks to reduce mortality by 2030 to levels experienced by the non-Indigenous population [40]. Addressing food insecurity among Aboriginal and Torres Strait Islanders by providing appropriate access to safe and nutritious foods has the potential to improve dietary behaviors that ultimately would contribute to increased life expectancy.

The ‘Closing the Gap’ strategy recognizes that the likelihood of co-morbidities is higher in the Aboriginal population when compared to the non-Indigenous population [41]. For example, about 38% of Aboriginal adults with either cardiovascular disease (CVD), diabetes or chronic kidney disease (CKD) had two or more health conditions compared to 26% of non-Indigenous people [2,41]. Coronary heart disease and diabetes are major contributors to the disease burden in Indigenous populations aged 45 years and over [2]. Poor dietary habits are known risk factors for poor health outcomes, with poor dietary habits contributing 50.1% towards the burden of CVD in Aboriginal populations [2,41]. Improving access to food is one factor that could affect the ability to choose a nutritious diet and improve dietary behaviors thereby improving health outcomes in Aboriginal and Torres Strait Islander population. 

### 4.2. Socio-Economic Characteristics Are Strongly Associated with Food Insecurity

The prevalence of food insecurity is not evenly spread throughout the population and we find socio-economic factors are strongly associated with food insecurity and that specific demographic groups report higher rates of food insecurity. In the model of food insecurity formulated here, socio-economic factors are viewed as moderating food insecurity through the degree of resource-constrained access to the food supply.

Specifically, we identify a number of population sub-groups with a food insecurity prevalence rate of around 1 in 3: Indigenous speakers in remote communities (37%), Aboriginal and Torres Strait Islander only households (28%), smokers (31%), persons in households with a large number of occupants (32%), non-married females (35%) and income earners in the lowest centile (31%). 

Differences in food insecurity status by socio-economic factors is consistent with studies in community dwelling non-Indigenous older Australians showing food insecurity differs by a range of socio-demographic risk factors, such as income, marital status, and smoker status [14,15,16]. The strength of socio-economic factors in explaining heightened food insecurity among Indigenous populations has also been noted elsewhere. For example, Pardilla et al. (2013), in their study of the Navajo Nation, note “Low socio-economic status, which is highly prevalent on the Navajo Nation and which we found to be significantly associated with food insecurity, must be considered in future endeavors to improve food security and decrease the risk of chronic disease” ([10] p. 64).

The strength of socio-economic factors as risk factors in experiencing food insecurity is concerning given the continued economic deprivation experienced by many Aboriginal and Torres Strait Islanders. For example, considerable disparities in socio-economic outcomes persist between non-Indigenous and Aboriginal persons across all age groups through lower levels of education, employment and living in areas with greater socio-economic disadvantage, while also being at considerable risk of experiencing interpersonal racism [5,42]. Not only do socio-economic factors provide greater access to the food supply (e.g., due to greater resources with which to purchase food), but these resources may also enable families to better cope with unexpected shocks (e.g., unexpected unemployment or disability) that could be a precursor to food insecurity.

### 4.3. Food Insecurity is High in Both Urban and Remote Settings

Results presented here also underscore differences in food insecurity risks between remote and non-remote areas of Australia. Interestingly, we find that non-English speaking persons in remote areas (37.3%) are at double the risk of exposure to food insecurity than English speakers in either remote (19%) or non-remote (18.7%) Australia. 

Part of this disparity may reflect the lack of detailed geographical measures on NATSINPAS due to confidentialization. That is, non-English speakers are more likely to be resident in particularly isolated areas of remote Australia—with limited access to affordable food sources and health facilities. Notwithstanding, a considerable literature has emerged on the disparity in food prices between urban, regional, and remote regions in Australia. In one study, a 47% difference in prices was reported between remote and urban supermarkets for healthier foods [43]. Solutions to this complex problem include improvements to freight and transport costs, as well as improving low levels of demand for nutritious foods [44]. Indeed, one of the key drivers of food choice in remote Aboriginal communities is poverty [45]. These transport and demand issues are exacerbated by the lack of availability and variety of healthier food choices and lack of locally grown food in remote areas of Australia [45,46]. Combining the high cost of food and high poverty levels in remote regions likely explains the increased likelihood of being food insecure for non-English speaking Aboriginal and Torres Strait Islanders. 

However, it was interesting to note that the likelihood of being food insecure did not differ between non- remote and remote English-speaking Aboriginal and Torres Strait Islanders with about 1 in 5 at risk of exposure to food insecurity. This is important as 75% of the Aboriginal and Torres Strait Islander population live in urban and regional areas [47]. Furthermore, projections of the Aboriginal and Torres Strait Islander population indicate future growth of the older population to be more prevalent in cities and regional areas. Between 2016 and 2026, the population aged 45 years and over is projected to grow by 36% in major cities, 39% in Inner and Outer regional areas and by 24% in remote and very remote areas of Australia [6]. These findings suggest that solutions to addressing food insecurity in older Aboriginal and Torres Strait Islander populations need to focus on all geographical locations. Indeed, recent efforts have tended to focus on food insecurity in remote areas, with less focus given to people living in urban areas [48,49]. 

Despite the focus of recent programs, a greater awareness of the linguistic and cultural needs in remote areas is required for effective solutions to food insecurity. In a systematic review of Aboriginal food and nutrition programs, Browne and colleagues (2018) conclude that “the most important factor determining success of Aboriginal and Torres Strait Islander food and nutrition programs is community involvement in (and ideally, control of) program development and implementation” [50]. As further argued by Bramwell et al. (2017), “given the sensitivity and shame often associated with food insecurity, more needs to be known about how health professionals can broach the issue to ensure dignity and cultural safety” ([48] p. 7). Herein lies a major problem for the implementation of food insecurity programs, as there is a considerable underrepresentation of Aboriginal and Torres Strait Islander people in the health sector—including at the programmatic design level [51]. 

### 4.4. Study Limitations

In interpreting our results, it is important to consider the study’s limitations. Our findings potentially underestimate the degree of food insecurity in this population for several reasons. Firstly, the NATSINPAS population is limited to Aboriginal and Torres Strait Islanders residing in private dwellings and excludes a range of other vulnerable populations such as the homeless, those in poor health living in care facilities or hospitals. Indeed, there is a considerable need for further research on food insecurity risk in non-community based settings, among both Indigenous and non-Indigenous populations [52]. Secondly, the measure of food insecurity we employ is primarily an indicator of food insecurity as it only addresses access to food by economic means and does not include physical or mobility access. For example, research has shown that along with financial barriers, storage, transportation, health, and functional barriers are associated with experiencing food insecurity [53,54]. Using a more comprehensive tool that addressed issues of anxiety, quality and quantity of food suggested the prevalence of food insecurity in non-Indigenous older adults was 13% compared to 2% resulting from responses to the single item tool [16]. Furthermore, qualitative research suggests that many older people engage in “precarious nutritional self-management strategies” that are important indicators of food insecurity, and are not captured in the measures used herein [55]. Specific to this population, access to and availability of skills to use traditional foods or ‘bush tucker’ is also likely to be an important component of overall food security [12]. The food insecurity of older Aboriginal and Torres Strait Islanders would undoubtedly be higher according to these broader definitions. Finally, the prevalence and severity of food insecurity may be cyclical and cannot be captured in cross sectional data [56,57]. Longitudinal data are necessary to measure complex movements in and out of food insecurity and unfortunately this data is not collected. Moreover, as these data are cross sectional, we cannot and do not draw a causal relationship between the independent variables and food insecurity. 

## 5. Conclusions

Noting these limitations, this paper was the first to examine food insecurity among older Aboriginal and Torres Strait Islanders. We found that food insecurity is experienced by a sizeable minority of older persons (1 in 5), that 41% of this group go without food consequently and that specific demographic groups are at a considerably heightened risk. In total, older Aboriginal and Torres Strait Islanders are at about a 5–7 fold risk of experiencing food insecurity relative to their non-Indigenous peers. 

These findings offer information with which to identify food insecure persons and to inform nutrition programs in place to improve health and wellbeing. Existing studies note the importance of involving Aboriginal and Torres Strait Islander people in the management, design, and implementation of such programs and considerably more needs to be done in this area. However, nutrition programs alone are likely to be ineffective. The strength of socio-economic factors in explaining the prevalence of food insecurity, suggest that policies must improve economic and social wellbeing (through the ‘Closing the Gap’ strategy) in tandem with targeted nutrition programs and policies aimed at providing an affordable, healthy food supply. Recent evaluation studies, unfortunately, show slow progress with the ‘Closing the Gap’ strategy, with improvements in some but not all health and economic outcomes for Aboriginal and Torres Strait Islanders over the past decade [58,59]. 

Our findings further suggest solutions to addressing food insecurity in older Aboriginal and Torres Strait Islander populations need to focus on all geographical locations—not just on remote Australia, which has been the focus to date. In both remote and non-remote Australia, greater awareness of linguistic and cultural needs as well as the involvement and management of programs for and by Aboriginal and Torres Strait Islanders is required to improve levels of food security. Addressing food insecurity through these means offers one pathway for reducing nutrition related disease and co-morbidities, thereby assisting the ‘Closing the Gap’ strategy to achieve its aim of improving health outcomes for Aboriginal and Torres Strait Islanders.

## Figures and Tables

**Table 1:** Food Insecurity (%) by Socio-Economic Characteristics, 2012–2013.

	Food Secure		Insecure: Depletion		Insecure: Depletion & Intake		Food Insecure ^1^	Unweighted *n* =
**Age**								
45–54	76.3	-	14.2	-	9.5	-	23.7	435
55–64	82.6		9.8		7.6		17.4	356
65–74	80.6		11.0		8.4		19.4	200
75+	89.7		8.7		1.6		10.3	71
**Self-Reported Health**								
Excellent or Good	81.4	-	9.9	-	8.7	-	18.6	667
Fair or Poor	76.1	*	16		7.9	*	23.9	395
**Language & Remoteness**								
Non-remote	81.3	-	10.6	-	8.1	-	18.7	451
Remote-English	80.9		11.6		7.4		19.0	374
Remote-Indigenous ^2^	62.7	***	25.1	***	12.2	***	37.3	236
**Household Income** ^3^								
Lowest 20%	69.1	-	16.7	-	14.3	-	31.0	472
20–40%	86.9	**	7.9		5.2	*	13.1	215
40–60%	90.5	***	5.0	**	4.5	*	9.5	109
60–80%	84.1	***	7.1	***	8.8	***	15.9	77
80–100%	94.7	***	5.3	***	0.0	***	5.3	57
Unknown	77.1		18.9		3.9		22.8	132
**Household Composition**								
Aboriginal only	72.1	-	15.6	-	11.9	-	27.5	779
Aboriginal and Non ^4^	92.6	***	5.4	***	2.0	***	7.4	283
**Smoker Status**								
Yes	68.8	-	16.1	-	15.1	-	31.2	420
No	86.9	***	9.4	*	3.7	**	13.1	642
**Household Size**								
1	75.1	-	14.6	-	10.3	-	24.9	422
2	88.3	**	7.5	*	4.2	*	11.7	413
3–4	72.8		14.1		13.2		27.3	176
5+	68.1	*	24.0	*	7.9		31.9	51
**Marital Status**								
Female, Unmarried	65.4	-	17.7	-	16.9	-	34.6	393
Female, Married	90.5	***	5.7	***	3.7	**	9.4	225
Male, Unmarried	79.3	*	13.6		7.0		20.6	247
Male, Married	87.3	***	9.7		9.7	***	19.4	197
**Non-school Qual ^5^**								
Yes	79.2	-	11.4	-	9.4	-	20.8	370
No	79.6	*	12.7		7.7		20.4	692
Weighted 45+ (%)	79.5		12.2		8.4		20.6	
Unweighted 45+ (%)	78.2		13.5		8.3		21.8	
Unweighted (n)	831		143		88		231	1062

^1^ Includes respondents indicating food insecurity with food depletion and those indicating food insecurity with food depletion and inadequate intake; ^2^ Indigenous language; ^3^ Equivalized household income (to allow for welfare comparisons across households of different sizes) placed into quintiles; ^4^ Non-Indigenous household members present; ^5^ Has received post-school education or training; *** *p* < 0.001, ** *p* < 0.01, * *p* < 0.05; - omitted category for tests of proportions. Weighted estimates.

**Table 2:** Ordered Logistic Regression Model of Food Insecurity, 2012–2013.

Covariate	Odds Ratio (OR)	95% CI ^1^	
**Age**			
45–54	1.00		
55–64	0.81	0.56, 1.16	
65–74	0.61	0.39, 0.96	*
75+	0.46	0.23, 0.91	*
**Marital Status**			
Female, Unmarried	1.00		
Female, Married	0.52	0.30, 0.89	*
Male, Unmarried	0.73	0.49, 1.08	
Male, Married	0.62	0.36, 1.05	+
**Labor Force Status**			
Employed	1.00		
Unemployed	1.58	1.05, 2.36	*
**Smoker Status**			
Yes	1.00		
No	0.61	0.44, 0.84	***
**Self-Reported Health**			
Excellent or Good	1.00		
Fair or Poor	1.53	1.10, 2.11	*
**Household Income ^2^**			
Lowest 20%	1.00		
20–40%	0.69	0.45, 1.06	+
40–60%	0.62	0.32, 1.19	
60–80%	0.24	0.08, 0.71	**
80–100%	0.12	0.02, 0.95	*
Unknown	0.89	0.56, 1.42	
**Language & Remoteness**			
Non-Remote	1.00		
Remote-English	0.99	0.68, 1.47	
Remote-Indigenous ^3^	1.57	1.01, 2.44	*
**Household Composition**			
Aboriginal only	1.00		
Aboriginal and Non ^4^	0.40	0.22, 0.69	***
**Household Size**			
1	1.00		
2	1.27	0.80, 2.01	
3–4	1.95	1.17, 3.25	**
5+	2.64	1.28, 5.46	**

^1^ 95% Confidence Interval for the Odds Ratio; ^2^ Equivalized household income (to allow for welfare comparisons across households of different sizes) placed into quintiles; ^3^ Indigenous language; ^4^ Non-Indigenous household members present; *** *p* < 0.001, ** *p* < 0.01, * *p* < 0.05, + *p* < 0.1.
